# Association of Physical Therapy Interventions With Long-term Opioid Use After Total Knee Replacement

**DOI:** 10.1001/jamanetworkopen.2021.31271

**Published:** 2021-10-27

**Authors:** Kosaku Aoyagi, Tuhina Neogi, Christine Peloquin, Maureen Dubreuil, Lee Marinko, James Camarinos, David T. Felson, Deepak Kumar

**Affiliations:** 1 Section of Rheumatology, Department of Medicine, Boston University School of Medicine, Boston, Massachusetts; 2 OptumLabs, Eden Prairie, Minnesota; 3 Department of Physical Therapy and Athletic Training, Boston University, Boston, Massachusetts

## Abstract

**Importance:**

Many individuals who undergo total knee replacement (TKR) become long-term opioid users after TKR. Associations of physical therapy (PT) interventions before or after TKR with long-term use of opioids are not known.

**Objectives:**

To evaluate associations of PT interventions before and after TKR with long-term opioid use after TKR.

**Design, Setting, and Participants:**

This cohort study used data from the OptumLabs Data Warehouse on 67 322 individuals aged 40 years or older who underwent TKR from January 1, 2001, to December 31, 2016, stratified by history of opioid use. The analyses for the study included data from January 1, 1999, to December 31, 2018.

**Exposures:**

Any PT interventions within 90 days before or after TKR, post-TKR PT dose as number of sessions (ie, 1-5, 6-12, and ≥13 sessions), post-TKR PT timing as number of days to initiation of care (ie, <30 days, 31-60 days, or 61-90 days after TKR), and post-TKR PT type (ie, active vs passive).

**Main Outcomes and Measures:**

The association of pre- and post-TKR PT with risk of long-term opioid use occurring more than 90 days after TKR was assessed using logistic regression while adjusting for confounders, including age, sex, race and ethnicity (Asian, Black, Hispanic, or White), obesity, type of insurance, geographical location, and physical and mental health comorbidities.

**Results:**

A total of 38 408 opioid-naive individuals (21 336 women [55.6%]; mean [SD] age, 66.2 [9.2] years) and 28 914 opioid-experienced individuals (18 426 women [63.7%]; mean [SD] age, 64.4 [9.3] years) were included. Receipt of any PT before TKR was associated with lower odds of long-term opioid use in the opioid-naive (adjusted odds ratio [aOR], 0.75 [95% CI, 0.60-0.95]) and opioid-experienced (aOR, 0.75 [95% CI, 0.70-0.80]) cohorts. Receipt of any post-TKR PT was associated with lower odds of long-term use of opioids in the opioid-experienced cohort (aOR, 0.75 [95% CI, 0.70-0.79]). Compared with 1 to 5 sessions of PT after TKR, 6 to 12 sessions (aOR, 0.82 [95% CI, 0.75-0.90]) and 13 or more sessions (aOR, 0.71 [95% CI, 0.65-0.77) were associated with lower odds in the opioid-experienced cohort. Compared with initiation of PT within 30 days after TKR, initiation 31 to 60 days or 61 to 90 days after TKR were associated with greater odds in the opioid-naive (31-60 days: aOR, 1.45 [95% CI, 1.19-1.77]; 61-90 days: aOR, 2.15 [95% CI, 1.43-3.22]) and opioid-experienced (31-60 days: aOR, 1.10 [95% CI, 1.02-1.18]; 61-90 days: aOR, 1.32 [95% CI, 1.12-1.55]) cohorts. Compared with passive PT, active PT was not associated with long-term opioid use in the opioid-naive (aOR, 1.00 [95% CI, 0.81-1.24]) or opioid-experienced (aOR, 0.99 [95% CI, 0.92-1.07]) cohorts.

**Conclusions and Relevance:**

This cohort study suggests that receipt of PT intervention before and after TKR, receipt of 6 or more sessions of PT care after TKR, and initiation of PT care within 30 days after TKR were associated with lower odds of long-term opioid use. These findings suggest that PT may help reduce the risk of long-term opioid use after TKR.

## Key Points

**Question:**

Are physical therapy (PT) interventions before and after total knee replacement (TKR) associated with lower risk of postoperative long-term use of opioids?

**Findings:**

In this cohort study that included 67 322 individuals who underwent TKR, receipt of any pre- and post-TKR PT care, receipt of more than 5 post-TKR PT sessions, and initiation of PT care within 30 days after TKR were associated with lower risk of long-term opioid use.

**Meaning:**

This study suggests that receipt of PT interventions may be associated with lower risk of long-term opioid use after TKR; these data may help inform effective dose and timing of PT interventions after TKR.

## Teaser

This cohort study evaluates associations of physical therapy interventions before and after total knee replacement with long-term use of opioids after total knee replacement among US adults.

## Introduction

Knee osteoarthritis is the most common form of arthritis worldwide, with pain as the primary symptom, causing reduced quality of life.^1,2,3,4^ Total knee replacement (TKR) is the only definitive therapy available to ameliorate pain and disability for those with severe end-stage knee osteoarthritis.^4^ More than 650 000 knee replacements were performed in the US in 2008,^4^ and 3.5 million procedures per year are expected by 2030.^5^ However, among those who undergo TKR, 20% to 30% of patients have postoperative persistent pain (defined as pain of at least 3 months’ duration that develops or increases in intensity after surgery),^6,7^ and a substantial proportion of those end up using opioids over the long term.^8,9^ It has been reported that 34.7% to 53.5% of patients who used opioids preoperatively and 5.0% to 8.2% patients who were naive to preoperative opioid use became long-term opioid users after TKR.^8,9^

Opioids are the primary pain reliever typically used to manage postoperative TKR pain acutely.^10^ However, opioid prescriptions after TKR are a common starting point for conversion to long-term opioid use,^8,9,11^ which is associated with greater risk of persistent postoperative pain in addition to the well-recognized risks of morbidity, mortality, and disability.^11^ Clearly, additional efforts are needed to reduce the use of opioids after TKR, but little guidance is available about efficacious and safe alternative options. Physical therapy (PT) interventions after TKR are effective in reducing pain and improving functional outcomes and, given these effects, they may be effective in reducing opioid use after TKR.^12^ This is, to our knowledge, unstudied. Furthermore, while there is agreement on the need for post-TKR rehabilitation supervised by physical therapists,^13^ there is not yet agreement on the timing and duration of rehabilitation after TKR, contributing to the variability in post-TKR PT interventions.^14,15^ Although active interventions (eg, exercise, gait training) are generally more effective than passive interventions (eg, TENS [transcutaneous electrical nerve stimulation], cold therapy) in reducing pain in people with knee osteoarthritis,^16,17^ information regarding the association of type of PT interventions with long-term opioid use, a surrogate for pain management, is lacking for patients undergoing TKR.^18^ “Prerehabilitation” prior to TKR is associated with benefits in postoperative pain, function, and length of stay,^19,20,21^ but the association with post-TKR long-term opioid use is not known.

We evaluated the association of PT interventions before and after TKR, relative to not receiving PT care, with long-term opioid use after TKR, including specific characteristics of post-TKR PT interventions (ie, dose, type, and timing of PT interventions). We hypothesized that any PT before and after TKR would be associated with a lower risk of long-term opioid use. Furthermore, we hypothesized that postoperatively, a higher dose of PT, earlier initiation of PT, and receipt of active PT would be associated with a lower risk of long-term opioid use.

## Methods

### Study Sample

We used data from the OptumLabs Data Warehouse, which includes deidentified medical and pharmacy claims, laboratory test results, and enrollment records for commercial insurance and Medicare Advantage enrollees. The database contains longitudinal health information on enrollees and patients, representing a diverse mixture of ages, races, ethnicities, and geographical regions across the US. Members in the database had full insurance coverage for physician, hospital, and prescription drug services.^22^ Race was categorized as Asian, Black, Hispanic, White, or unknown and derived using vendor-developed algorithms that rely on the individual’s name and geographical location.^22^ Because this study involved analysis of preexisting, deidentified data, the Boston Medical Center and Boston University Medical Campus institutional review board granted exemption from approval. This study followed the Strengthening the Reporting of Observational Studies in Epidemiology (STROBE) reporting guideline.

We identified individuals aged 40 years or older who underwent TKR between January 1, 2001, and December 31, 2016, based on *Current Procedural Terminology* (*CPT*) procedure codes and *International Classification of Diseases, Ninth Revision* (*ICD-9*) and *International Statistical Classification of Diseases and Related Health Problems, Tenth Revision* (*ICD-10*) diagnosis codes (eTable 1 in the Supplement). The date of TKR was considered the index date. Included individuals were also required to have continuous coverage 24 months prior to TKR and during the follow-up period after TKR (details of the follow-up period are in the outcomes subsection below), with the availability of both medical and pharmacy claims. We excluded individuals with any knee surgery, rheumatoid arthritis, or cancer within 24 months prior to the index date.

Individuals included in the study were categorized as those with prior opioid use (opioid experienced) and those without prior opioid use (opioid naive).^8,9,23,24^ Opioid-experienced individuals were defined as individuals with 2 or more filled opioid prescriptions within 12 months prior to TKR, while opioid-naive users were individuals with no opioid prescription within the 12 months prior to TKR. Individuals with a single opioid prescription in the 12 months prior to TKR were excluded from the main analyses to avoid inclusion of single prescriptions for standard acute medical or dental issues; these individuals were included in a separate sensitivity analysis.^25^

### Exposures

Our primary exposure variables were occurrence of any visit with a physical therapist before and after TKR, with PT episode of care (EOC) identified using *CPT* codes (eTable 2 in the Supplement). Individuals with 1 or more outpatient or inpatient PT *CPT* code within 90 days prior to TKR were considered as having received a pre-TKR PT intervention (referred to as pre-TKR PT). Patients with 1 or more PT *CPT* codes at an outpatient facility within 90 days after TKR were considered as having received a post-TKR PT intervention (referred to as post-TKR PT).

To define the attributes of PT interventions after TKR (ie, dose, type, and timing), we identified the first outpatient PT EOC that started within 90 days after surgery. A post-TKR PT EOC was considered to have ended when the EOC was followed by an absence of any PT claims for 12 weeks afterward. The 12-week period was not considered to be part of the EOC; rather, it was included in the period eligible for evaluation of the outcome. Definitions of these exposures are summarized in eTable 3 in the Supplement. Active PT interventions were defined as 50% or more of codes (eTable 2 in the Supplement) being active interventions during the post-TKR PT EOC.^26^

### Outcomes

Our outcome interest was long-term opioid use, defined as 90 days’ worth or more of filled opioid prescriptions during the outcome assessment periods.^27^ We identified oral and enteral formulations of opioid prescriptions. A rheumatologist investigator (M.D.) selected the opioids (eTable 4 in the Supplement).

**Figure 1.**
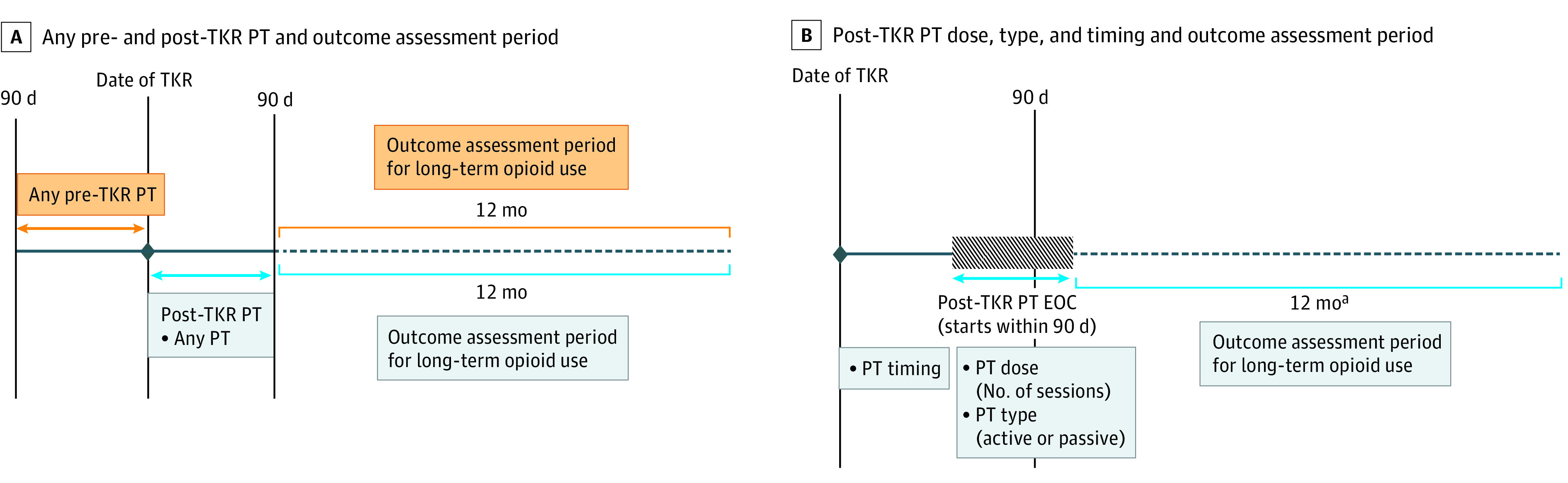
Study Timeline A, Any pre– and post–total knee replacement (TKR) physical therapy (PT) and outcome assessment period. B, Post-TKR PT dose, type, and timing and outcome assessment period. ^a^The PT episode of care (EOC) has to start within 90 days of TKR but can end at any time after TKR. Hence, the outcome assessment period varies across patients owing to differences in post-TKR PT EOC start date and duration.

The outcome assessment period for pre-TKR and post-TKR PT interventions was 12 months after the first 90 days after TKR to exclude any opioid use in the immediate postoperative period (Figure 1A). The outcome assessment period for dose, timing, and type of post-TKR PT was the 12-month period after the end of the post-TKR PT EOC (Figure 1B). These definitions of long-term opioid use after TKR represent a time in which normal surgical recovery is expected and is more conservative than the definition of postsurgical persistent pain by the International Association for the Study of Pain.^28,29^

### Potential Confounders

All analyses were adjusted for potential confounders including age, sex, race and ethnicity (Asian, Black, Hispanic, or White; included given disparities in provision of opioids and PT),^30,31^ obesity (yes or no), type of insurance (commercial or Medicare Advantage), geographical location, and physical and mental health comorbidities. Geographical locations were Midwest, Northeast, South, and West. Comorbidities were identified throughout the 24 months prior to the index date using the Elixhauser Comorbidity Index.^32^ Physical comorbidities were included as the count of physical comorbidities (eg, congestive heart failure, paralysis).^32^ The following were excluded for calculating the total count of physical comorbidities: obesity, alcohol abuse, drug abuse, psychoses, and depression.^33^ Obesity was entered into the model as a separate covariate. Psychological comorbidities were identified using the Chronic Condition Data Warehouse algorithm created by the Centers for Medicare & Medicaid Services.^34^ Each psychological comorbidity was entered into models as an individual covariate,^33^ with exceptions of bipolar disorder, posttraumatic stress disorder, and schizophrenia disorder, which were combined because of the potential collinearity between these covariates. Furthermore, calendar year was added as a covariate for all models to account for secular trends, while pre-TKR PT was added as a covariate for post-TKR PT models.

### Other Descriptive Data

We identified use of nonsteroidal anti-inflammatory drugs and medications for other common chronic musculoskeletal conditions in these individuals within 24 months prior to TKR. Use of nonsteroidal anti-inflammatory drugs was defined as prescription of 1 or more of the following medications: celecoxib, diclofenac, diflunisal, fenoprofen, flurbiprofen, ibuprofen, indomethacin, ketoprofen, ketorolac, meloxicam, naproxen, nabumetone, oxaprozin, phenylbutazone, rofecoxib, salsalate, sulindac, tolmetin, and valdecoxib. We used *ICD-9* and *ICD-10* codes to identify fibromyalgia, low back pain, neck pain, and shoulder pain as the other long-term musculoskeletal conditions.

### Statistical Analysis

Analyses for the study included data from January 1, 1999, to December 31, 2018. Descriptive data were summarized for key variables in the opioid-experienced and opioid-naive cohorts. We evaluated the associations of pre-TKR PT and post-TKR PT (ie, any PT intervention, and dose, timing, and type of PT intervention) with long-term opioid use in the 12-month period after the first 90 days after TKR (for any pre- and post-TKR PT interventions) or after the PT EOC ended (for dose, timing, and type of PT intervention) in the opioid-experienced and opioid-naive cohorts in separate logistic regression models. For sensitivity analyses, we repeated the analyses in the cohort of those who had a single opioid prescription 12 months prior to the index date. All analyses were conducted using SAS, version 9.4 (SAS Institute Inc). The statistical significance level was set at a 2-sided α level of .05 for all analyses.

## Results

**Figure 2.**
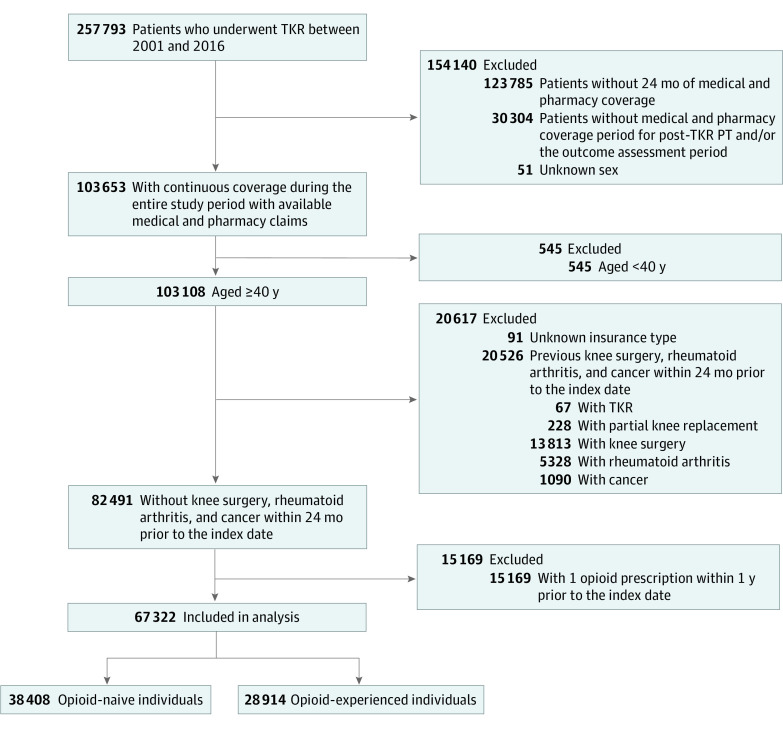
Participant Flow Diagram PT indicates physical therapy; and TKR, total knee replacement.

We identified 257 793 patients who underwent TKR from 2001 to 2016, of whom 67 322 patients met our inclusion criteria (Figure 2): 38 408 (21 336 women [55.6%]; 7262 [18.9%] with obesity; mean [SD] age, 66.2 [9.2] years) were opioid naive and 28 914 (18 426 women [63.7%]; 8242 [28.5%] with obesity; mean [SD] age, 64.4 [9.3] years) were opioid experienced (Table 1). A total of 15 169 individuals (8924 women [58.8%]; 3395 [22.4%] with obesity; mean [SD] age, 65.5 [9.0] years) had 1 opioid prescription within 12 months prior to the index date (eTable 5 in the Supplement). The number of individuals included for each analysis is indicated in the footnotes of Table 2 and Table 3 because it varied depending on the outcome assessment period.

**Table 1.** Cohort Characteristics

Characteristic	Patients, No. (%)	
	Opioid naive (n = 38 408)	Opioid experienced (n = 28 914)
Age, mean (SD), y	66.2 (9.2)	64.4 (9.3)
Sex		
Female	21 336 (55.6)	18 426 (63.7)
Male	17 072 (44.4)	10 488 (36.3)
Obesity	7262 (18.9)	8242 (28.5)
Race		
Asian	621 (1.6)	299 (1.0)
Black	3078 (8.0)	3395 (11.7)
Hispanic	1774 (4.6)	1395 (4.8)
White	31 798 (82.8)	23 108 (79.9)
Missing	1137 (3.0)	717 (2.5)
US Region		
Midwest	15 052 (39.2)	9817 (34.0)
Northeast	4291 (11.2)	2030 (7.0)
South	13 466 (35.1)	12 764 (44.1)
West	5585 (14.5)	4290 (14.8)
Missing	14 (0.04)	13 (0.04)
Insurance type		
Commercial	25 805 (67.2)	19 143 (66.2)
Medicare Advantage	12 603 (32.8)	9771 (33.8)
Pre-TKR opioid prescriptions, median (IQR)^a^	NA	4 (2-9)
NSAID use^b^	18 148 (47.3)	20 674 (71.5)
Fibromyalgia	2672 (7.0)	4455 (15.4)
Low back pain	11 241 (29.3)	15 438 (53.4)
Neck pain	6161 (16.0)	7844 (27.1)
Shoulder pain	2601 (6.8)	3522 (12.2)
Elixhauser physical comorbidities, mean (SD), No.^c^	2.5 (2.0)	3.3 (2.4)
ADHD	156 (0.4)	253 (0.9)
Depression	4406 (11.5)	7035 (24.3)
Bipolar disorder, schizophrenia disorder, or PTSD	464 (1.2)	875 (3.0)
Substance use disorder	104 (0.3)	577 (2.0)
Alcohol use disorder	239 (0.6)	414 (1.4)
Anxiety	1918 (5.0)	3423 (11.8)
Dementia	129 (0.3)	106 (0.4)

Abbrevations: ADHD, attention-deficit/hyperactivity disorder; IQR, interquartile range; NA, not applicable; NSAID, nonsteroidal anti-inflammatory drug; PTSD, posttraumatic stress disorder; TKR, total knee replacement.

^a^ Pre-TKR opioid prescriptions: filled opioid prescriptions within 12 months prior to TKR.

^b^ Including celecoxib, diclofenac, diflunisal, fenoprofen, flurbiprofen, ibuprofen, indomethacin, ketoprofen, ketorolac, meloxicam, naproxen, nabumetone, oxaprozin, phenylbutazone, rofecoxib, salsalate, sulindac, tolmetin, and valdecoxib.

^c^ Only physical comorbidities from the original Elixhauser Comorbidity Index were included in this count.

**Table 2.** Association of Any Pre-TKR PT and Any Post-TKR PT With Long-term Opioid Use After TKR Among Opioid-Naive and Opioid-Experienced Patients

Characteristic	Opioid naive^a^			Opioid experienced^b^		
	Patients, No. (%)	Long-term opioid use, No. (%)	Adjusted OR (95% CI)	Patients, No. (%)	Long-term opioid use, No. (%)	Adjusted OR (95% CI)
Any pre-TKR PT						
Yes	4627 (12.8)	87 (1.9)	0.75 (0.60-0.95)	4408 (16.3)	1368 (31.0)	0.75 (0.70-0.80)
No	31 524 (87.2)	766 (2.4)	1 [Reference]	22 690 (83.7)	8129 (35.8)	1 [Reference]
Any post-TKR PT						
Yes	27 911 (77.2)	631 (2.3)	0.89 (0.75-1.04)	19 827 (73.2)	6457 (32.6)	0.75 (0.70-0.79)
No	8240 (22.8)	222 (2.7)	1 [Reference]	7271 (26.8)	3040 (41.8)	1 [Reference]

Abbreviations: OR, odds ratio; PT, physical therapy; TKR, total knee replacement.

^a^ Of the 38 408 individuals in the opioid-naive cohort, 36 151 had data available over 15 months after TKR (ie, 90-day period to exclude any opioid use in the immediate postoperative period and subsequent 12 months of the outcome assessment period).

^b^ Of the 28 914 individuals in the opioid-experienced cohort, 27 098 had data available over 15 months after TKR (ie, 90-day period to exclude any opioid use in the immediate postoperative period and subsequent 12 months of the outcome assessment period).

**Table 3.** Association of Dose, Timing, and Type of Post-TKR PT With Long-term Opioid Use After TKR Among Opioid-Naive and Opioid-Experienced Patients

Characteristic	Opioid naive^a^			Opioid experienced^b^		
	Patients, No. (%)	Long-term opioid use, No. (%)	Adjusted OR (95% CI)	Patients, No. (%)	Long-term opioid use, No. (%)	Adjusted OR (95% CI)
Post-TKR PT dose (No. of sessions)						
≥13	12 202 (47.2)	249 (2.0)	0.82 (0.67-1.01)	8516 (46.7)	2525 (29.7)	0.71 (0.65-0.77)
6-12	7354 (28.5)	161 (2.2)	0.90 (0.72-1.14)	4952 (27.2)	1668 (33.7)	0.82 (0.75-0.90)
1-5	6285 (24.3)	150 (2.4)	1 [Reference]	4771 (26.2)	1735 (36.4)	1 [Reference]
Post-TKR PT timing, d						
61-90	635 (2.5)	28 (4.4)	2.15 (1.43-3.22)	704 (3.9)	299 (42.5)	1.32 (1.12-1.55)
31-60	5214 (20.2)	152 (2.9)	1.45 (1.19-1.77)	4278 (23.5)	1497 (35.0)	1.10 (1.02-1.18)
≤30	19 992 (77.4)	380 (1.9)	1 [Reference]	13 257 (72.7)	4132 (31.2)	1 [Reference]
Post-TKR PT type						
Active	20 612 (79.8)	443 (2.1)	1.00 (0.81-1.24)	14 288 (78.3)	4642 (32.5)	0.99 (0.92-1.07)
Passive	5229 (20.2)	117 (2.2)	1 [Reference]	3951 (21.7)	1286 (32.5)	1 [Reference]

Abbreviations: OR, odds ratio; PT, physical therapy; TKR, total knee replacement.

^a^ Of the 38 408 individuals in the opioid-naive cohort, 25 841 received post-TKR PT and had data available over the 12 months’ outcome assessment period after the end of the episode of care.

^b^ Of the 28 914 individuals in the opioid-experienced cohort, 18 239 received post-TKR PT and had data available over the 12 months’ outcome assessment period after the end of the episode of care.

### Pre-TKR PT Intervention and Long-term Opioid Use

During the study period, 9035 of 63 249 participants (14.3%) received pre-TKR PT interventions (Table 2). Overall, 853 of 36 151 participants in the opioid-naive cohort (2.4%) and 9497 of 27 098 participants in the opioid-experienced cohort (35.0%) had long-term opioid use. Receipt of any pre-TKR PT intervention was associated with lower odds of long-term opioid use compared with those who did not receive any pre-TKR PT intervention in both the opioid-naive cohort (adjusted odds ratio [aOR], 0.75 [95% CI, 0.60-0.95]) and the opioid-experienced cohort (aOR, 0.75 [95% CI, 0.70-0.80]).

### Post-TKR PT Intervention and Long-term Opioid Use

A total of 47 738 of 63 249 participants (75.5%) received outpatient PT interventions within 90 days after surgery (Table 2). Median post-TKR PT EOC duration was 46 days and median post-TKR PT EOC intensity was 0.35 visits per week for the opioid-naive cohort and 48 days and 0.33 visits per week, respectively, for the opioid-experienced cohort. Receipt of post-TKR PT was associated with lower odds of long-term opioid use in the opioid-experienced cohort (aOR, 0.75 [95% CI, 0.70-0.79]). The difference in the opioid-naive cohort was not statistically significant (aOR, 0.89 [95% CI, 0.75-1.04]) (Table 2).

### Characteristics of Post-TKR PT Interventions and Long-term Opioid Use

Overall, 560 of 25 841 participants in the opioid-naive cohort (2.2%) and 5928 of 18 239 participants in the opioid-experienced cohort (32.5%) had long-term opioid use (Table 3). A total of 11 056 of 44 080 participants (25.1%) received 1 to 5 sessions of PT, 12 306 of 44 080 participants (27.9%) received 6 to 12 sessions of PT, and 20 718 of 44 080 participants (47.0%) received 13 or more sessions of PT. In the opioid-experienced cohort, receipt of 6 to 12 sessions of PT (aOR, 0.82 [95% CI, 0.75-0.90]) and 13 or more sessions of PT (aOR, 0.71 [95% CI, 0.65-0.77]) was associated with lower odds of long-term opioid use compared with receipt of 1 to 5 sessions (Table 3). The findings for the opioid-naïve group were not statistically significant (6-12 sessions: aOR, 0.90 [95% CI, 0.72-1.14]; ≥13 sessions; aOR, 0.82 [95% CI, 0.67-1.01]).

Compared with care initiation within 30 days after TKR, care initiation 31 to 60 days or 61 to 90 days after TKR was associated with greater odds of long-term opioid use for the opioid-naive cohort (31-60 days: aOR, 1.45 [95% CI, 1.19-1.77]; 61-90 days: aOR, 2.15 [95% CI, 1.43-3.22]) and for the opioid-experienced cohort (31-60 days: aOR, 1.10 [95% CI, 1.02-1.18]; 61-90 days: aOR, 1.32 [95% CI, 1.12-1.55]) (Table 3). Compared with passive PT, active PT was not associated with long-term opioid use in the opioid-naive (aOR, 1.00 [95% CI, 0.81-1.24]) or opioid-experienced (aOR, 0.99 [95% CI, 0.92-1.07]) cohorts.

### Sensitivity Analyses

Sensitivity analyses were conducted among those with history of a single opioid prescription during the 12-month period prior to TKR. Results were either similar to those of the opioid-experienced cohort or in between the opioid-naive and opioid-experienced cohorts (eTable 6 in the Supplement).

## Discussion

Both pre-TKR and post-TKR PT interventions were associated with lower odds of long-term opioid use after TKR. Greater number of PT intervention sessions and earlier initiation of outpatient PT care after TKR were associated with lower odds of long-term opioid use; however, the type of post-TKR PT intervention (active vs passive) was not associated with the outcome. These results suggest that PT interventions may be associated with lower risk of long-term opioid use after TKR and provide insights for developing guidelines on effective dose and timing of PT interventions after TKR.

We noted that 14.3% of individuals received any pre-TKR PT intervention, similar to 10.5% reported in a prior study.^35^ We also observed that most individuals (75.5%) received post-TKR outpatient PT care within 90 days of TKR, consistent with other studies.^36^ The lower odds of long-term opioid use after TKR with pre-TKR PT interventions complements findings of prior studies that reported benefits associated with pre-TKR rehabilitation for postoperative pain, function, and length of hospital stay.^19,37,38^ However, a recent consensus statement from the Enhanced Recovery After Surgery Society recommended against preoperative PT interventions for patients with TKR.^18^ The recommendations were made based on the moderate-quality evidence from meta-analyses that show small and short-term associations of pre-TKR PT interventions with postoperative outcomes; these outcomes did not include long-term opioid use.^20,39,40^ We also noted lower odds of long-term opioid use with any post-TKR PT intervention in the opioid-experienced group. We focused on outpatient PT care after TKR, given that all patients generally receive acute PT during their post-TKR hospitalization.^36^

A total of 47.0% of participants received 13 or more PT sessions after TKR; prior studies have reported a range from 10.1 to 14.5 sessions.^14,41^ Greater number of PT sessions has been associated with better pain and function after TKR.^36,41^ Our findings in a large real-world cohort provide data regarding an additional benefit associated with post-TKR PT, with higher PT dose being associated with lower odds of long-term opioid use in the opioid-experienced cohort; the opioid-naive cohort exhibited a similar, although nonsignificant, trend.

Initiation of outpatient PT care within 1 month after TKR was associated with lower odds of long-term opioid use after TKR than later initiation in both the opioid-experienced and opioid-naive cohorts. In prior studies, earlier initiation of PT was associated with better function and lower pain; however, opioid use was not studied.^41,42^ The American Physical Therapy Association identified the need for research to investigate the optimal timing of post-TKR rehabilitation.^43^ Our findings suggest that initiating outpatient PT care within 30 days after TKR may be associated with a lower risk of long-term opioid use.

We did not observe an association between active vs passive PT interventions after TKR and long-term opioid use. In people with knee osteoarthritis, active PT interventions are more effective than passive interventions.^16,17^ However, associations of the type of PT intervention with outcomes in people with TKR are relatively understudied. A meta-analysis concluded that the level of evidence regarding the association of passive vs active PT interventions with opioid use was low.^44^ The recent American Physical Therapy Association guidelines recommend both passive and active PT interventions after TKR, but are mostly related to the immediate postoperative phase.^43^ Hence, further work is needed to understand the relative association of active and passive PT interventions with outcomes in outpatient settings after TKR.

Similar to prior studies, 32.5% to 35.0% of opioid-experienced individuals and 2.2% to 2.4% of opioid-naive individuals in our study had long-term opioid use after TKR.^8,9,23,24,45,46,47,48^ Although the incidence of long-term opioid use among opioid-naive individuals is lower than among those with prior opioid use, these results are concerning given the large number of TKR surgical procedures performed annually in the US and projected increases in this surgery in the future.^5^ Our results suggest that pre-TKR and post-TKR PT interventions are associated with lower risk of long-term opioid use regardless of prior opioid use.

### Limitations

This study has some limitations. First, we did not adjust for opioid use within 90 days after surgery because opioids are commonly prescribed for acute postoperative pain at discharge.^49,50^ Second, the outcome assessment periods for the pre-TKR and post-TKR PT exposures were slightly different because the post-TKR PT EOC could extend beyond 90 days after TKR, and we aimed to examine the risk of long-term opioid use after completion of post-TKR PT. Third, while patients with inadequate improvement in pain and function are usually referred to PT, we cannot rule out the possibility that some patients who received pre-TKR and post-TKR PT were simply those who were less likely to be treated with opioids (and vice versa—that those taking opioids may be less inclined to undergo PT); thus our findings could reflect confounding by indication. A randomized clinical trial would be required to disentangle these issues. Fourth, we did not adjust for access to PT care across different states. Because regulations regarding access to PT can change over time, it was not possible to adjust for this variation in a study such as ours comprising data from a 15-year period, although we accounted for calendar year to attempt to address secular trends. We were also unable to account for physician-owned vs nonphysician-owned PT practices, which can have an association with the number and type of PT sessions that patients receive.^36^ Fifth, we did not have information on functional status during acute care after TKR that may be associated with the use of PT. Sixth, we adjusted for obesity instead of body mass index because not all individuals within the claims database have body mass index data; thus, there may be residual confounding, although the magnitude of residual confounding that would remain after accounting for obesity status is unclear. Seventh, we are unable to comment on more granular insights regarding whether PT interventions are associated with use of lower dosages of opioids, as we did not evaluate morphine milligram equivalent doses.

## Conclusions

This cohort study found that both pre-TKR and post-TKR PT interventions, and certain characteristics of post-TKR PT, such as greater number of sessions and earlier initiation, were associated with lower long-term use of opioids during the year after TKR, when pain and function have generally stabilized. Assessment of long-term opioid use after TKR may be a pertinent end point to consider in recommendations regarding PT interventions before and after TKR.

## Notes

Supplement. **eTable 1.** Total Knee Replacement Codes Used as Inclusion Criteria **eTable 2.** *CPT* Codes for Active and Passive Physical Therapy Interventions **eTable 3.** A List of Exposures and Outcome **eTable 4.** Included and Excluded Opioids **eTable 5.** Descriptive Data for Patients With One Opioid Prescription Within 12 Months Prior to the Index Date **eTable 6.** Association of Dose, Timing, and Type of Post-TKR PT with Long-Term Opioid Use After TKR Among Patients With One Opioid Prescription Within 12 Months Prior to the Index Date

## References

[1] Neogi T. The epidemiology and impact of pain in osteoarthritis. Osteoarthritis Cartilage. 2013;21(9):1145-1153. doi:10.1016/j.joca.2013.03.018 23973124 PMC3753584

[2] Vos T, Flaxman AD, Naghavi M, . Years lived with disability (YLDs) for 1160 sequelae of 289 diseases and injuries 1990-2010: a systematic analysis for the Global Burden of Disease Study 2010. Lancet. 2012;380(9859):2163-2196. doi:10.1016/S0140-6736(12)61729-2 23245607 PMC6350784

[3] GBD 2016 Causes of Death Collaborators. Global, regional, and national age-sex specific mortality for 264 causes of death, 1980-2016: a systematic analysis for the Global Burden of Disease Study 2016. Lancet. 2017;390(10100):1151-1210. doi:10.1016/S0140-6736(17)32152-9 28919116 PMC5605883

[4] Carr AJ, Robertsson O, Graves S, . Knee replacement. Lancet. 2012;379(9823):1331-1340. doi:10.1016/S0140-6736(11)60752-6 22398175

[5] Kurtz S, Ong K, Lau E, Mowat F, Halpern M. Projections of primary and revision hip and knee arthroplasty in the United States from 2005 to 2030. J Bone Joint Surg Am. 2007;89(4):780-785. doi:10.2106/00004623-200704000-00012 17403800

[6] Beswick AD, Wylde V, Gooberman-Hill R, Blom A, Dieppe P. What proportion of patients report long-term pain after total hip or knee replacement for osteoarthritis? a systematic review of prospective studies in unselected patients. BMJ Open. 2012;2(1):e000435. doi:10.1136/bmjopen-2011-000435 22357571 PMC3289991

[7] Wylde V, Beswick A, Bruce J, Blom A, Howells N, Gooberman-Hill R. Chronic pain after total knee arthroplasty. EFORT Open Rev. 2018;3(8):461-470. doi:10.1302/2058-5241.3.180004 30237904 PMC6134884

[8] Politzer CS, Kildow BJ, Goltz DE, Green CL, Bolognesi MP, Seyler TM. Trends in opioid utilization before and after total knee arthroplasty. J Arthroplasty. 2018;33(7S):S147, 153. doi:10.1016/j.arth.2017.10.060 29198871

[9] Goesling J, Moser SE, Zaidi B, . Trends and predictors of opioid use after total knee and total hip arthroplasty. Pain. 2016;157(6):1259-1265. doi:10.1097/j.pain.0000000000000516 26871536 PMC4868627

[10] Stasiowska MK, Ng SC, Gubbay AN, Cregg R. Postoperative pain management. Br J Hosp Med (Lond). 2015;76(10):570-575. doi:10.12968/hmed.2015.76.10.570 26457937

[11] Pizzi LT, Toner R, Foley K, . Relationship between potential opioid-related adverse effects and hospital length of stay in patients receiving opioids after orthopedic surgery. Pharmacotherapy. 2012;32(6):502-514. doi:10.1002/j.1875-9114.2012.01101.x 22570188

[12] Artz N, Elvers KT, Lowe CM, Sackley C, Jepson P, Beswick AD. Effectiveness of physiotherapy exercise following total knee replacement: systematic review and meta-analysis. BMC Musculoskelet Disord. 2015;16:15. doi:10.1186/s12891-015-0469-6 25886975 PMC4333167

[13] Westby MD, Brittain A, Backman CL. Expert consensus on best practices for post-acute rehabilitation after total hip and knee arthroplasty: a Canada and United States Delphi study. Arthritis Care Res (Hoboken). 2014;66(3):411-423. doi:10.1002/acr.22164 24023047

[14] Oatis CA, Johnson JK, DeWan T, Donahue K, Li W, Franklin PD. Characteristics of usual physical therapy post–total knee replacement and their associations with functional outcomes. Arthritis Care Res (Hoboken). 2019;71(9):1171-1177. doi:10.1002/acr.23761 30281207 PMC6447481

[15] Warren M, Shireman TI. Geographic variability in discharge setting and outpatient postacute physical therapy after total knee arthroplasty: a retrospective cohort study. Phys Ther. 2018;98(10):855-864. doi:10.1093/ptj/pzy077 29945184

[16] Kolasinski SL, Neogi T, Hochberg MC, . 2019 American College of Rheumatology/Arthritis Foundation Guideline for the management of osteoarthritis of the hand, hip, and knee. Arthritis Rheumatol. 2020;72(2):220-233. doi:10.1002/art.41142 31908163 PMC10518852

[17] Bannuru RR, Osani MC, Vaysbrot EE, . OARSI guidelines for the non-surgical management of knee, hip, and polyarticular osteoarthritis. Osteoarthritis Cartilage. 2019;27(11):1578-1589. doi:10.1016/j.joca.2019.06.011 31278997

[18] Wainwright TW, Gill M, McDonald DA, . Consensus statement for perioperative care in total hip replacement and total knee replacement surgery: Enhanced Recovery After Surgery (ERAS) Society recommendations. Acta Orthop. 2020;91(1):3-19. doi:10.1080/17453674.2019.1683790 31663402 PMC7006728

[19] Santa Mina D, Clarke H, Ritvo P, . Effect of total-body prehabilitation on postoperative outcomes: a systematic review and meta-analysis. Physiotherapy. 2014;100(3):196-207. doi:10.1016/j.physio.2013.08.008 24439570

[20] Moyer R, Ikert K, Long K, Marsh J. The value of preoperative exercise and education for patients undergoing total hip and knee arthroplasty: a systematic review and meta-analysis. JBJS Rev. 2017;5(12):e2. doi:10.2106/JBJS.RVW.17.00015 29232265

[21] Hoogeboom TJ, Oosting E, Vriezekolk JE, . Therapeutic validity and effectiveness of preoperative exercise on functional recovery after joint replacement: a systematic review and meta-analysis. PLoS One. 2012;7(5):e38031. doi:10.1371/journal.pone.0038031 22675429 PMC3364996

[22] OptumLabs. Accessed July 1, 2020. https://www.optumlabs.com/

[23] Bedard NA, Pugely AJ, Westermann RW, Duchman KR, Glass NA, Callaghan JJ. Opioid use after total knee arthroplasty: trends and risk factors for prolonged use. J Arthroplasty. 2017;32(8):2390-2394. doi:10.1016/j.arth.2017.03.014 28413136

[24] Brummett CM, Janda AM, Schueller CM, . Survey criteria for fibromyalgia independently predict increased postoperative opioid consumption after lower-extremity joint arthroplasty: a prospective, observational cohort study. Anesthesiology. 2013;119(6):1434-1443. doi:10.1097/ALN.0b013e3182a8eb1f 24343289 PMC3867739

[25] Johnston SS, Ammann E, Scamuffa R, . Association of body mass index and osteoarthritis with healthcare expenditures and utilization. Obes Sci Pract. 2020;6(2):139-151. doi:10.1002/osp4.398 32313672 PMC7156818

[26] Fritz JM, Cleland JA, Brennan GP. Does adherence to the guideline recommendation for active treatments improve the quality of care for patients with acute low back pain delivered by physical therapists? Med Care. 2007;45(10):973-980. doi:10.1097/MLR.0b013e318070c6cd 17890995

[27] Martin BC, Fan MY, Edlund MJ, Devries A, Braden JB, Sullivan MD. Long-term chronic opioid therapy discontinuation rates from the TROUP Study. J Gen Intern Med. 2011;26(12):1450-1457. doi:10.1007/s11606-011-1771-0 21751058 PMC3235603

[28] Werner MU, Kongsgaard UEI. I: defining persistent post-surgical pain: is an update required? Br J Anaesth. 2014;113(1):1-4. doi:10.1093/bja/aeu012 24554546

[29] Macrae WA, Davies HTO. Chronic postsurgical pain. In: Crombie IK, ed. *Epidemiology of Pain*. IASP Press; 1999;125-142.

[30] Sandstrom R, Bruns A. Disparities in access to outpatient rehabilitation therapy for African Americans with arthritis. J Racial Ethn Health Disparities. 2017;4(4):599-606. doi:10.1007/s40615-016-0263-7 27400913

[31] Jin Y, Solomon DH, Franklin PD, . Patterns of prescription opioid use before total hip and knee replacement among US Medicare enrollees. Osteoarthritis Cartilage. 2019;27(10):1445-1453. doi:10.1016/j.joca.2019.05.023 31251985 PMC6751003

[32] Elixhauser A, Steiner C, Harris DR, Coffey RM. Comorbidity measures for use with administrative data. Med Care. 1998;36(1):8-27. doi:10.1097/00005650-199801000-00004 9431328

[33] Kazis LE, Ameli O, Rothendler J, . Observational retrospective study of the association of initial healthcare provider for new-onset low back pain with early and long-term opioid use. BMJ Open. 2019;9(9):e028633. doi:10.1136/bmjopen-2018-028633 31542740 PMC6756340

[34] Chronic Condition Data Warehouse, Centers for Medicare & Medicaid Services. Condition categories. Accessed September 17, 2021. https://www2.ccwdata.org/web/guest/condition-categories

[35] Snow R, Granata J, Ruhil AV, Vogel K, McShane M, Wasielewski R. Associations between preoperative physical therapy and post-acute care utilization patterns and cost in total joint replacement. J Bone Joint Surg Am. 2014;96(19):e165. doi:10.2106/JBJS.M.01285 25274793

[36] Pua YH, Seah FJ, Poon CL, Tan JW, Liaw JS, Chong HC. Association between rehabilitation attendance and physical function following discharge after total knee arthroplasty: prospective cohort study. Osteoarthritis Cartilage. 2017;25(4):462-469. doi:10.1016/j.joca.2016.10.020 27810379

[37] McKay C, Prapavessis H, Doherty T. The effect of a prehabilitation exercise program on quadriceps strength for patients undergoing total knee arthroplasty: a randomized controlled pilot study. PM R. 2012;4(9):647-656. doi:10.1016/j.pmrj.2012.04.012 22698852

[38] Calatayud J, Casaña J, Ezzatvar Y, Jakobsen MD, Sundstrup E, Andersen LL. High-intensity preoperative training improves physical and functional recovery in the early post-operative periods after total knee arthroplasty: a randomized controlled trial. Knee Surg Sports Traumatol Arthrosc. 2017;25(9):2864-2872. doi:10.1007/s00167-016-3985-5 26768606

[39] Wang L, Lee M, Zhang Z, Moodie J, Cheng D, Martin J. Does preoperative rehabilitation for patients planning to undergo joint replacement surgery improve outcomes? systematic review and meta-analysis of randomised controlled trials. BMJ Open. 2016;6(2):e009857. doi:10.1136/bmjopen-2015-009857 26839013 PMC4746481

[40] Chen H, Li S, Ruan T, Liu L, Fang L. Is it necessary to perform prehabilitation exercise for patients undergoing total knee arthroplasty: meta-analysis of randomized controlled trials. Phys Sportsmed. 2018;46(1):36-43. doi:10.1080/00913847.2018.1403274 29125384

[41] Brennan GP, Fritz JM, Houck LTCKM, Hunter SJ. Outpatient rehabilitation care process factors and clinical outcomes among patients discharged home following unilateral total knee arthroplasty. J Arthroplasty. 2015;30(5):885-890. doi:10.1016/j.arth.2014.12.013 25765128

[42] Liebs TR, Herzberg W, Rüther W, Haasters J, Russlies M, Hassenpflug J; Multicenter Arthroplasty Aftercare Project. Multicenter randomized controlled trial comparing early versus late aquatic therapy after total hip or knee arthroplasty. Arch Phys Med Rehabil. 2012;93(2):192-199. doi:10.1016/j.apmr.2011.09.011 22196125

[43] Jette DU, Hunter SJ, Burkett L, ; American Physical Therapy Association. Physical therapist management of total knee arthroplasty. Phys Ther. 2020;100(9):1603-1631. doi:10.1093/ptj/pzaa099 32542403 PMC7462050

[44] Tedesco D, Gori D, Desai KR, . Drug-free interventions to reduce pain or opioid consumption after total knee arthroplasty: a systematic review and meta-analysis. JAMA Surg. 2017;152(10):e172872. doi:10.1001/jamasurg.2017.2872 28813550 PMC5831469

[45] Naik BI. Redefining opioid use patterns after surgical procedures: why a new paradigm is critical. JAMA Netw Open. 2020;3(6):e209457. doi:10.1001/jamanetworkopen.2020.9457 32584405

[46] Dunn LK, Yerra S, Fang S, . Incidence and risk factors for chronic postoperative opioid use after major spine surgery: a cross-sectional study with longitudinal outcome. Anesth Analg. 2018;127(1):247-254. doi:10.1213/ANE.0000000000003338 29570151 PMC6487073

[47] Brummett CM, Waljee JF, Goesling J, . New persistent opioid use after minor and major surgical procedures in US adults. JAMA Surg. 2017;152(6):e170504. doi:10.1001/jamasurg.2017.0504 28403427 PMC7050825

[48] Sun EC, Darnall BD, Baker LC, Mackey S. Incidence of and risk factors for chronic opioid use among opioid-naive patients in the postoperative period. JAMA Intern Med. 2016;176(9):1286-1293. doi:10.1001/jamainternmed.2016.3298 27400458 PMC6684468

[49] Calcaterra SL, Yamashita TE, Min SJ, Keniston A, Frank JW, Binswanger IA. Opioid prescribing at hospital discharge contributes to chronic opioid use. J Gen Intern Med. 2016;31(5):478-485. doi:10.1007/s11606-015-3539-4 26553336 PMC4835366

[50] Lloret-Linares C, Lopes A, Declèves X, . Challenges in the optimisation of post-operative pain management with opioids in obese patients: a literature review. Obes Surg. 2013;23(9):1458-1475. doi:10.1007/s11695-013-0998-8 23700237

