# Supplementary material for: Association of Physical Therapy Interventions With Long-term Opioid Use After Total Knee Replacement
Source: JAMA Netw Open. 2021 Oct 27;4(10):e2131271. doi: 10.1001/jamanetworkopen.2021.31271 (PMC8552057; doi:10.1001/jamanetworkopen.2021.31271)
Supplement: Supplement. — eTable 1. Total Knee Replacement Codes Used as Inclusion Criteria eTable 2. CPT Codes for Active and Passive Physical Therapy Interventions eTable 3. A List of Exposures and Outcome eTable 4. Included and Excluded Opioids eTable 5. Descriptive Data for Patients With One Opioid Prescription Within 12 Months Prior to the Index Date eTable 6. Association of Dose, Timing, and Type of Post-TKR PT with Long-Term Opioid Use After TKR Among Patients With One Opioid Prescription Within 12 Months Prior to the Index Date [file jamanetwopen-e2131271-s001.pdf]

# Supplementary Online Content

Aoyagi K, Neogi T, Peloquin C, et al. Association of physical therapy interventions with long-term opioid use after total knee replacement. *JAMA Netw Open.* 2021;4(10):e2131271. doi:10.1001/jamanetworkopen.2021.31271

**eTable 1.** Total Knee Replacement Codes Used as Inclusion Criteria

**eTable 2.** *CPT* Codes for Active and Passive Physical Therapy Interventions

**eTable 3.** A List of Exposures and Outcome

**eTable 4.** Included and Excluded Opioids

**eTable 5.** Descriptive Data for Patients With One Opioid Prescription Within 12 Months Prior to the Index Date

**eTable 6.** Association of Dose, Timing, and Type of Post-TKR PT With Long-Term Opioid Use After TKR Among Patients With One Opioid Prescription Within 12 Months Prior to the Index Date

This supplementary material has been provided by the authors to give readers additional information about their work.

© 2021 Aoyagi K et al. *JAMA Network Open.*

**eTable 1.** Total Knee Replacement Codes Used as Inclusion Criteria

| **Total Knee Replacement Codes** |
| --- |
| CPT |
| 27447 |
| ICD-10-PCS |
| 0SRC069, 0SRC06A, 0SRC06Z, 0SRC07Z, 0SRC0J9, 0SRC0JA, 0SRC0JZ, 0SRC0KZ, 0SRC0L9, 0SRC0LA, 0SRC0LZ, 0SRD069, 0SRD06A, 0SRD06Z, 0SRD07Z, 0SRD0J9, 0SRD0JA, 0SRD0JZ, 0SRD0KZ, 0SRD0L9, 0SRD0LA, 0SRD0LZ |
| ICD-9-CM |
| 81.54 |

© 2021 Aoyagi K et al. *JAMA Network Open.*

**eTable 2.** *CPT* Codes for Active and Passive Physical Therapy Interventions

| ***CPT* Codes** | **Physical Therapy** |
|---|---|
| **Active** | |
| 97110 | THERAPEUTIC EXERCISES |
| 97112 | NEUROMUSCULAR REEDUCATION |
| 97113 | AQUATIC THERAPY/EXERCISES |
| 97116 | GAIT TRAINING THERAPY |
| 97530 | THERAPEUTIC ACTIVITIES |
| 97535 | SELF CARE MANAGEMENT TRAINING |
| **Passive** | |
| 97010 | HOT OR COLD PACKS THERAPY |
| 97012 | MECHANICAL TRACTION THERAPY |
| 97014 | ELECTRIC STIMULATION THERAPY |
| 97016 | VASOPNEUMATIC DEVICE THERAPY |
| 97018 | PARAFFIN BATH THERAPY |
| 97022 | WHIRLPOOL THERAPY |
| 97024 | DIATHERMY EG, MICROWAVE |
| 97026 | INFRARED THERAPY |
| 97028 | ULTRAVIOLET THERAPY |
| 97032 | ELECTRICAL STIMULATION |
| 97033 | ELECTRIC CURRENT THERAPY |
| 97034 | CONTRAST BATH THERAPY |
| 97035 | ULTRASOUND THERAPY |
| 97036 | HYDROTHERAPY |
| 97124 | MASSAGE THERAPY |
| 97140 | MANUAL THERAPY 1/> REGIONS |
| G0283 | ELECTRICAL STIMULATION, OTHER THAN WOUND CARE, AS PART OF A THERAPY PLAN OF CARE |
| **Evaluation** | |
| 97001 | PT EVALUATION |
| 97161 | PHYSICAL THERAPY EVALUATION LOW COMPLEX 20 MINS |
| 97162 | PHYSICAL THERAPY EVALUATION MODERATE COMPLEX 30 MINS |
| 97163 | PHYSICAL THERAPY EVALUATION HIGH COMPLEX 45 MINS |

© 2021 Aoyagi K et al. *JAMA Network Open.*

**eTable 3.** A List of Exposures and Outcome

| Exposures | Definition |
|---|---|
| *Pre-TKR PT* | |
| Any PT | One or more PT evaluation or treatment within 90 days before TKR |
| *Post-TKR PT* | |
| Any PT | One or more PT evaluation or treatment within 90 days after TKR |
| PT Dose | Number of PT visits on unique dates within the first PT EOC initiated within 90 days post-TKR. Categorized as 1-5, 6-12, 13+ sessions. |
| PT Timing | Number of days to initiation of PT within 90 days post-TKR. Categorized as <30 days, 31-60 days, 61-90 days. |
| PT Type | Active PT was defined as >=50% of CPT codes in the first PT EOC being active PT interventions (see supplementary eTable 3 for CPT codes used to define active and passive PT) |
| *Chronic opioid use was the outcome for all models, defined as >+ 90days worth of filled opioid prescriptions over the outcome assessment periods (see Figure 1 for the assessment period)* | |

© 2021 Aoyagi K et al. *JAMA Network Open.*

**eTable 4.** Included and Excluded Opioids

| |
|---|
| **Included opioids: opioid prescriptions of oral/enteral formulations of the following:** |
| fentanyl, codeine, hydrocodone, oxycodone, meperidine, hydromorphone, morphine, methadone, oxymorphone, propoxyphene, levorphanol, levomethadyl, pentazocine, butorphanol, tapentadol, and tramadol. |
| **Excluded opioids** |
| Combinations of opioids with medications commonly used for cough, nasal congestion, and headache; excluded opioid combinations were those containing pseudoephedrine, guaifenesin, pheynylephrine, bromopheniramine, pyrilamine, bulbital and caffeine. |

© 2021 Aoyagi K et al. *JAMA Network Open.*

**eTable 5.** Descriptive Data for Patients With One Opioid Prescription Within 12 Months Prior to the Index Date

| | **Patients, No. (%)** |
|---|---|
| | One Opioid (15,169) |
| Age (years), mean (SD) | 65.5 (9.0) |
| Female | 8,924 (58.8%) |
| Obesity | 3,395 (22.4%) |
| Race | |
| Asian | 190 (1.3%) |
| Black | 1,437 (9.5%) |
| Hispanic | 743 (4.9%) |
| White | 12,363 (81.5%) |
| Missing | 436 (2.9%) |
| US Region | |
| Midwest | 5,305 (35.0%) |
| Northeast/Missing | 1,294 (8.5%) |
| South | 6,208 (40.9%) |
| West | 2,362 (15.6%) |
| Insurance Type | |
| Commercial | 10,444 (68.9%) |
| Medicare Advantage | 4,725 (31.1%) |
| Pre-TKR Opioid Prescriptions^, median (IQR) | 1 (1-1) |
| NSAID use | 9,451 (62.3%) |
| Fibromyalgia | 1,285 (8.5%) |
| Low Back Pain | 5,608 (37.0%) |
| Neck Pain | 2,990 (19.7%) |
| Shoulder Pain | 1,385 (9.1%) |
| Elixhauser physical comorbidities, mean (SD) | 2.7 (2.1) |

© 2021 Aoyagi K et al. *JAMA Network Open.*

| | |
|---|---|
| ADHD | 73 (0.5%) |
| Depression | 2,269 (15.0%) |
| Bipolar Disorder/Schizophrenia Disorder/PTSD | 179 (1.2%) |
| Substance Use Disorder | 42 (0.3%) |
| Alcohol Use Disorder | 117 (0.8%) |
| Anxiety | 897 (5.9%) |
| Dementia | 41 (0.3%) |

^Pre-TKR Opioid Prescriptions: filled opioid prescriptions within 12 months prior to TKR
NSAID: Nonsteroidal anti-inflammatory drugs (including celecoxib, diclofenac, diflunisal, fenoprofen, flurbiprofen, ibuprofen, indomethacin, ketoprofen, ketorolac, meloxicam, naproxen, nabumetone, oxaprozin, phenylbutazone, rofecoxib, salsalate, sulindac, tolmetin, and valdecoxib)
ADHD: attention deficit hyperactivity disorder
PTSD: post-traumatic stress disorder

© 2021 Aoyagi K et al. *JAMA Network Open.*

**eTable 6.** Association of Dose, Timing, and Type of Post-TKR PT With Long-Term Opioid Use After TKR Among Patients with One Opioid Prescription Within 12 Months Prior to the Index Date

| | | One Opioid Prescription | | |
|---|---|---|---|---|
| | | **Patients, No. (%)[a]** | **Long-term Opioid Use, No. (%)** | **Adjusted OR (95% CI)** |
| **Any pre-TKR PT** | Yes | 2128 | 65 (3.1%) | 0.75 (0.58, 0.98) |
| | No | 12082 | 484 (4.0%) | 1.0 |
| **Any post-TKR PT** | Yes | 11136 | 404 (3.6%) | 0.82 (0.67, 1.00) |
| | No | 3074 | 145 (4.7%) | 1.0 |
| **Post-TKR PT Dose (# sessions)** | 13+ | 5019 | 198 (3.9%) | 1.00 (0.78, 1.28) |
| | 6-12 | 2863 | 75 (2.6%) | 0.65 (0.48, 0.89) |
| | 1-5 | 2479 | 100 (4.0%) | REF |
| **Post-TKR PT Timing (days)** | 61-90 | 277 | 19 (6.9%) | 1.92 (1.17, 3.17) |
| | 31-60 | 2099 | 82 (3.9%) | 1.13 (0.87, 1.46) |
| | 0-30 | 7985 | 272 (3.4%) | REF |
| **Post-TKR PT Type** | Active | 8116 | 297 (3.7%) | 1.13 (0.87, 1.47) |
| | Passive | 2245 | 76 (3.4%) | REF |

[a] Of the 15,169 individuals with one opioid prescription within 12 months prior to the index date, 14,210 had data available over 15-months following TKR for any pre- and post-TKR PT analyses (i.e., 90 day period to exclude any opioid use in the immediate postoperative period and subsequent 12-months of outcome assessment period), and 10,361 received post-TKR PT and had data available over the 12-month outcome assessment period after the end of the episode of care for post-TKR PT dose, timing and type analyses.

© 2021 Aoyagi K et al. *JAMA Network Open.*
